# Improving Hand Function of Severely Impaired Chronic Hemiparetic Stroke Individuals Using Task-Specific Training With the ReIn-Hand System: A Case Series

**DOI:** 10.3389/fneur.2018.00923

**Published:** 2018-11-07

**Authors:** Carolina Camona, Kevin B. Wilkins, Justin Drogos, Jane E. Sullivan, Julius P. A. Dewald, Jun Yao

**Affiliations:** ^1^Department of Physical Therapy and Human Movement Sciences, Northwestern University, Chicago, IL, United States; ^2^Department of Biomedical Engineering, Northwestern University, Chicago, IL, United States; ^3^Department of Physical Medicine & Rehabilitation, Northwestern University, Chicago, IL, United States

**Keywords:** stroke, upper extremities, rehabilitation, functional electric stimulation (FES), hand function, task-practice

## Abstract

**Purpose:** In this study, we explored whether improved hand function is possible in poststroke chronic hemiparetic individuals with severe upper limb motor impairments when they participate in device-aided task-specific practice.

**Subjects:** Eight participants suffering from chronic stroke (>1-year poststroke, mean: 11.2 years) with severely impaired upper extremity movement (Upper Extremity Subscale of the Fugl-Meyer Motor Assessment (UEFMA) score between 10 and 24) participated in this study.

**Methods:** Subjects were recruited to participate in a 20-session intervention (3 sessions/7 weeks). During each session, participants performed 20–30 trials of reaching, grasping, retrieving, and releasing a jar with the assistance of a novel electromyography-driven functional electrical stimulation (EMG-FES) system.

This EMG-FES system allows for Reliable and Intuitive use of the Hand (called ReIn-Hand device) during multi-joint arm movements. Pre-, post-, and 3-month follow-up outcome assessments included the UEFMA, Cherokee McMaster Stroke Assessment, grip dynamometry, Box and Blocks Test (BBT), goniometric assessment of active and passive ranges of motion (ROMs) of the wrist and the metacarpophalangeal flexion and extension (II, V fingers), Nottingham Sensory Assessment–Stereognosis portion (NSA), and Cutaneous Sensory Touch Threshold Assessment.

**Results:** A nonparametric Friedman test of differences found significant changes in the BBT scores (χ^2^ = 10.38, *p* < 0.05), the passive and active ROMs (χ^2^ = 11.31, *p* < 0.05 and χ^2^ = 12.45, *p* < 0.01, respectively), and the NSA scores (χ^2^ = 6.42, *p* < 0.05) following a multi-session intervention using the ReIn-Hand device.

**Conclusions:** These results suggest that using the ReIn-Hand device during reaching and grasping activities may contribute to improvements in gross motor function and sensation (stereognosis) in individuals with chronic severe UE motor impairment following stroke.

## Introduction

Stroke is the second most common cause of mortality and the third most common cause of disability worldwide (1, 2). More than two-thirds of people who have had a stroke have difficulties with arm function, which contributes considerably in limiting the ability to perform activities of daily living (ADLs) (3, 4). Though various studies have reported positive outcomes following multiple types of interventions in more mildly impaired individuals (5, 6), regaining hand function in individuals with moderate-to-severe impairments still remains a challenge. This is largely due to impairments, such as the loss of volitional finger extension (7, 8), muscle coactivation (7), involuntary coupling of wrist and finger flexion with certain shoulder and elbow movements (9), and somatosensory deficits (10).

Several studies have suggested that repetitive task-specific training can improve upper extremity (UE) function (11–14) in mildly impaired stroke survivors when the practice is functionally relevant and of sufficient intensity. Intervention-induced gains have been reported for up to 6 months after intervention (15). In particular, interventions focusing on reach and grasp movements have been shown to be relevant because these movements are essential for ADLs and are viewed by subjects as high priority rehabilitative goals (16, 17). This approach has often been used in individuals in both the acute and subacute stage (18–20) and with mild-to-moderate impairments after stroke (6, 18, 21).

There is limited research targeting chronic stroke individuals with severely impaired UE. These individuals are less able to participate in task-specific training because of minimal volitional activation of the impaired arm (16). Furthermore, during ADLs, concurrent use of hand and arm are required. However, the presence of the flexion synergy after stroke (22–24), coupled with shoulder abduction with elbow/wrist and fingers flexion (9), decreases the ability to generate volitional or functional electrical stimulation (FES)-assisted finger extension while lifting against gravity (25, 26). This creates a major challenge to rehabilitation clinicians and limits opportunities for this population to participate in programs focused on hand recovery (16).

The purpose of this study is to determine the effect of device-assisted task-specific training on hand motor function and sensation (stereognosis and cutaneous sensory touch threshold) in individuals with chronic stroke and severe UE impairment. An electromyography-driven functional electrical stimulation (EMG-FES) with an intelligent detection software that detects the hand opening intention even with the presence of flexion synergies was used to assist the hand opening while subjects were performing required reaching and grasping tasks. We expected that by training a functional activity that involves arm-lifting, reaching and grasping, retrieving and releasing, poststroke participants with severely impaired UE would improve their arm/hand motor function and sensation.

Some parts of the results from various assessments [i.e., pre- to post-changes in an active range of motion (AROM) and Box and Blocks Test (BBT)] have been briefly reported in a previous publication (27) that focused on brain plasticity introduced by this ReIn-Hand assisted reaching and grasping intervention. Compared to the previous publication, this paper provides a complete overall report on various intervention-induced clinical changes.

## Methods

### Subjects

Eight adults (2 females, 6 males, mean age/range: 63.5/59–70 years) with chronic (mean time since stroke 11.2 years) UE hemiparesis resulting from a unilateral stroke participated in this study. All subjects completed the intervention protocol and before intervention (pre-), after intervention (post-), and 3-month follow-up assessments.

Inclusion criteria for this study included the following: (1) severe UE impairments with Upper Extremity Subscale of the Fugl-Meyer Motor Assessment (UEFMA) scores between 10 and 24 (28); (2) moderate to severe hand impairment with the Stage of Hand section of the Chedoke-McMaster Stroke Assessment (CMSA-H) (29) scores between 1 and 4, (3) at least 1 year after stroke; (4) the ability to open the hand, with a distance between the thumb and the index finger >4 cm, with the assistance of FES; (5) not pregnant or planning to become pregnant; and (6) the ability to provide informed consent. Exclusion criteria included the following: (1) inability to follow three step commands (30); (2) elbow flexion contracture >30°; (3) fixed finger/wrist flexion contracture >50°; (4) inability to attain 90° of passive shoulder flexion; (5) inability to sit more than 2 h (self-report); (6) any acute inflammatory or chronic painful conditions in the UE; (7) Botox injection/chemo-denervation within the last 6 months; (8) presence of cardiac pacemaker; (9) presence of a brainstem and or a cerebellar lesion; and (10) current participation in other interventions/studies. Participants' demographic information is illustrated in Table 1.

**Table 1:** Baseline characteristics.

**Characteristics**	**Total *N* = 8**
Age in years: mean (SD)	63.5 (4)
Years since Onset: mean (median, SD)	11.2 (9, 6.7)
Sex
Female	2
Male	6
Side of Hemiparesis
Right	6
Left	2
UE FMA Range (median, IQR)	10-24 (19.5, 11.5)
Chedoke-MacMaster-Hand: median (IQR)	3 (0.5)
Concordance
Concordant	4
Discordant	4
Sensory Impairment, number of subjects /8
SWMT	5/8
NSA Stereognosis	7/8

*Concordant, The paretic limb is the dominant limb; Discordant, The paretic limb is the non-dominant limb; UEFM, Upper Extremity Fugl-Meyer Assessment (Max score 66); IQR, Interquartile range; SWMT, Cutaneous Sensory Touch Threshold using Semmes-Weinstein Monofilaments (normal detection threshold: 2.83 filament, 0.05 grams) NSA Stereognosis Sensory Impairment ≤ 19*.

### Study settings and intervention

The study took place in a university research laboratory. All participants were recruited from the Clinical Neuroscience Research Registry hosted by the Shirley Ryan AbilityLab (former RIC) and Northwestern University. The Institutional Review Board at the Northwestern University approved the protocol for this study. Written informed consent was obtained from all subjects prior to testing.

### Device

“ReIn-Hand” is a recently developed EMG-FES device that uses the combination of an EMG collection unit (Avatar physiological recorder, Electrical Geodesics, Inc., Eugene, OR, United States), an intelligent detection software “the ReIn-HAND platform,” and an electrical stimulator (Empi 300, Vista, CA, United States) (31). The ReIn-Hand platform wirelessly and simultaneously measures surface EMG activities from eight upper limb muscles, including deltoid, biceps brachii, triceps, extensor communis digitorum, extensor carpi radialis, flexor digitorum profundus, flexor carpi radialis, and abductor pollicis. The device uses subject-dependent coherence-based notch filter to increase the signal-to-noise ratio of the collected EMG signals (32); it then uses the mean absolute value, zero crossing, slope sign changes, waveform length values (33) to perform real-time detection of hand opening with or without activation of the shoulder/elbow muscles during functional upper limb motor tasks (31). Once hand opening is detected, a signal is sent to trigger the electrical stimulator to assist paretic hand opening. In all the subjects, including those with abnormal synergistic muscle activity and spasticity, the average detection accuracies were >90% (31). The stimulation electrodes were placed over finger/wrist extensors; the stimulation was set with the following parameters: amplitude sufficient for maximal hand opening without discomfort, biphasic waveform, frequency 50 ± 20%, 300 μs pulse width, and duration time of 3 s.

### Intervention

**Figure 1:**
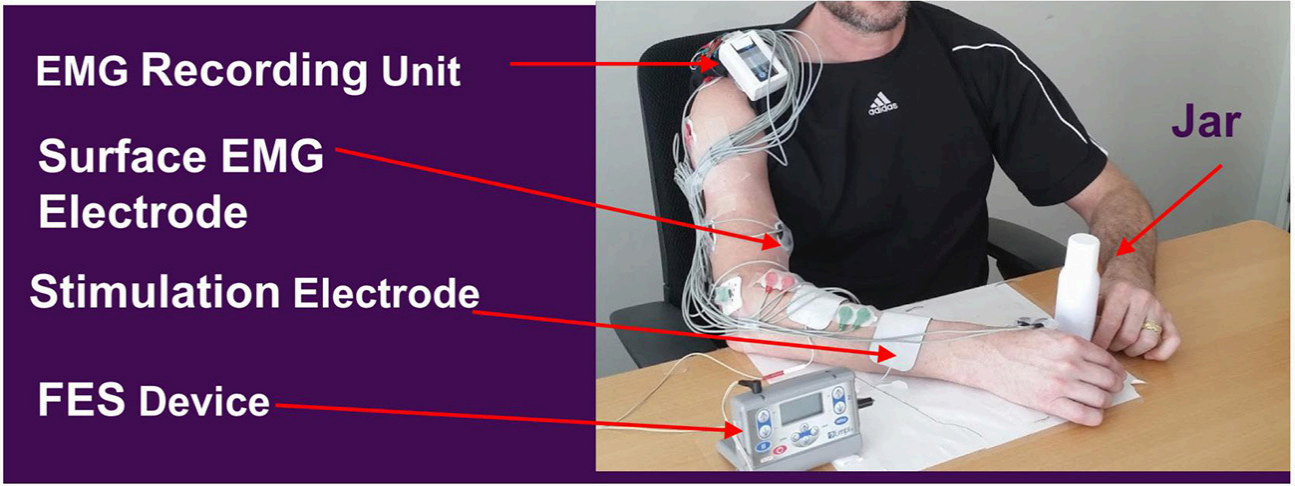
Rein-Hand device and the experimental set up. FES parameters: Amplitude sufficient for maximal hand opening without discomfort, biphasic waveform, frequency 50 Hz ± 20%, and 300 μs pulse width, and duration time 3 s. Adapted from Wilkins et al. (27).

Subjects participated in a 1.5–2.5-h session, 3 times per week, for 7 weeks. At the beginning of each session, muscles in the paretic arm/hand were stretched for about 10 min. The recording and stimulating electrodes were placed and the stimulation intensity was adjusted to allow for a maximal hand opening without discomfort. Each participant was seated in front of a height-adjustable table with a plastic jar (weight = 30 g, diameter = 4 or 5 cm, height = 13.5 cm) placed in front of their trunk, in line with the middle of their body (sagittal plane). Training consisted of 20–30 trials (approximately 1.0 h) of the following activities: (1). reaching forward toward the jar placed on a tabletop, (2). attempting to open the hand and activating finger/wrist extensor muscles to trigger the ReIn-Hand device in assisting the opening of the paretic hand, (3). grasping the jar, (4). bringing the jar toward their body and placing it on a table, and (5). releasing the jar. Hand opening and releasing were aided by the ReIn-Hand device. All the subjects were instructed not to fight the stimulation once the FES was successfully triggered; instead, they were asked to adjust their strategies to maximize the FES-assisted hand opening (e.g., either relaxing or opening the hand at a submaximal level). The diameter and weight of the jar and the distance/height to reach it were increased to make the task progressively more challenging, yet the task was set to allow each participant to complete the task and trials (Figure 1). For instance, the weight of the jar was gradually increased by 50–150 g, or more forearm supination during grasping and releasing of the jar was required; reaching distance were increased by 15 cm to achieve the full length use of the paretic arm, and height were increased from 0 to 10 cm. Also, a similar jar, with a large diameter (5 cm), was used as the participants' ability to open their hands improved. In order to avoid fatigue, a resting time of no <1 min was provided between each trials. The hemiparetic arm and hand were also stretched between trials to effectively elicit hand opening with the EMG-FES device.

### Outcome measures

Participants were evaluated by a research physical therapist before intervention (pre-assessment), after intervention (post-assessment), and at 3-month follow-up assessment. The BBT (34–37) was used as the assessment for activity measure. Meanwhile, the Upper Extremity Subscale of the Fugl-Meyer Motor Assessment (UEFMA) (28, 38), CMSA-H (29), grip strength (39, 40) (Jamar Technologies Hydraulic Hand Dynamometer 5030J1), the passive ROM (PROM) measured at the wrist and the II and V metacarpophalangeal joints, and AROM measured at the II and V metacarpophalangeal joints were used as the clinical assessment of motor impairment. Grip strength was measured in kilograms (Kgs), and we calculated a ratio between the paretic and nonparetic hand. The PROM and AROM were measured manually with a goniometer, where 0° was defined as the neutral position in the wrist and the fingers (0°s between flexion and extension) (41), negative values were indicated by flexion, and positive values were indicated by extension. Lastly, the Cutaneous Sensory Touch Threshold using Semmes-Weinstein Monofilaments (42, 43) and the Nottingham Sensory Assessment–Stereognosis portion (NSA) (44–46) were used for clinical assessments of sensory impairment.

### Data processing and statistical methods

The PROM measurements were averaged across three joints and reported as a single measure of the hand combining wrist and fingers. The AROM measurements were also averaged across joints and reported as a single measure of the fingers.

Statistics were performed using Matlab (2016a). The effects of the intervention were assessed with a nonparametric Friedman test. To obtain conditional to statistically significant (*p* < 0.05) values, *post-hoc* Wilcoxon signed-rank tests with Dunn-Sidak corrections for multiple comparisons were performed to further evaluate the impact of the intervention. Results are reported as significant (*p* < 0.05).

## Results

Results of the statistical analysis is summarized in Table 2, and individual data on all the clinical assessments at pre, post and 3-month follow-up is reported in Table 3.

**Table 2:** Outcomes at Baseline, Post and 3-Month Follow up.

**Clinical assessments**			**Session**		**Result**
		**Pre**	**Post**	**3 Month follow-up**	**Chi-square (p)**
Activity	BBT^*^	0 (0.75)	2 (5)	0 (3)	10.38 (*p =* 0.006)
Motor Impairment	PROM^*^	20.67 (25)	30.83 (40)	22.50 (37.5)	12.45 (*p =* 0.002)
	AROM^*^	0 (0)	5 (14)	4.50 (4)	11.31 (*p =* 0.03)
	CMSA-H	3(0.75)	3 (0)	3 (0)	3 (*p =* 0.22)
	Grip Ratio: P/NP	0.13 (0.36)	0.14 (0.43)	0.16 (0.36)	4.71 (*p =* 0.09)
	UEFMA	19.5 (11.5)	19 (11)	19 (11.5)	4.33 (*p =* 0.12)
Sensory Impairment	NSA^*^	15.5 (6)	18 (4)	19 (8)	6.42 (*p =* 0.04)
	SWMT	2.83 (1.31)	2.83 (1.31)	2.83 (0)	2.66 (*p =* 0.26)

*Values are the median and interquartile range in parenthesis for Pre, Post and Follow up session. Results Chi-Square and (P value)*.

*BBT, Box and Blocks Test; PROM, Passive Range of Motion; AROM, Active Range of Motion; CMSA-H, Chedoke-McMaster Stage of Hand section; P/NP, Paretic/Non Paretic; UEFM, Upper Extremity Fugl-Meyer; NSA, Nottingham-Stereognosis; SWMT, Cutaneous Sensory Touch Threshold using Semmes-Weinstein (SW) monofilament testing*.

* *Significant p < 0.05*.

Table 3Clinical assessments.

**Table:** 

**Motor impairment clinical assessment scores**															
	**CMSA-H**			**Grip ratio**			**PROM W-II-V**			**AROM II-V**			**UE FM**		
**S**	**Pre**	**Post**	**3 M**	**Pre**	**Post**	**3 M**	**Pre**	**Post**	**3 M**	**Pre**	**Post**	**3 M**	**Pre**	**Post**	**3 M**
	**P**	**P**	**P**	**P/NP**	**P/NP**	**P/NP**	**AVE**	**AVE**	**AVE**	**AVE**	**AVE**	**AVE**	**AVE**	**AVE**	**AVE**
S1	2	3	3	0.24	0.3	0.3	33.33	58.33	33.33	−20	11	3.5	23	23	23
S2	3	3	3	0.15	0.2	0.2	3.33	8.33	−20.67	0	5	5	11	12	11
S3	2	3	2	0.09	0.1	0.0	10.50	19	31.67	0	0	0	17	17	13
S4	3	3	3	0.10	0.1	0.1	23.33	40.00	28.33	0	17.5	5	10	11	11
S5	3	3	3	0.60	0.7	0.3	55.00	55.67	55.00	0	2.5	3	24	24	23
S6	3	3	3	0.07	0.1	0.1	20.00	33.33	19.33	0	1.5	0	13	13	12
S7	3	3	3	0.11	0.1	0.1	−13.33	−5.00	−9.00	0	5	4	24	24	24
S8	4	4	4	0.52	0.6	0.6	13.33	28.33	8.33	38.5	55	52.5	22	21	21

**Table:** 

**Activity measure scores**				**Sensory impairment clinical assessment scores**								
**BBT**				**NSA**						**SWMT**		
				**Pre**		**Post**		**3M**				
**S**	**Pre**	**Post**	**3 M**	**P**	**NP**	**P**	**NP**	**P**	**NP**	**Pre**	**Post**	**3 M**
S1	0	6	4	19	20	19	20	19	20	2.83	3.61	2.83
S2	1	3	0	18	19	18	20	19	20	2.83	2.83	2.83
S3	0	1	0	20	20	20	20	20	20	2.83	2.83	2.83
S4	0	1	0	14	19	18	20	20	20	2.83	2.83	2.83
S5	0	0	0	13	20	16	19	16	19	2.83	2.83	2.83
S6	0	0	0	17	20	20	20	20	20	3.61	2.83	2.83
S7	0	3	0	12	20	15	20	12	20	4.31	4.31	4.31
S8	11	13	8	0	20	2	20	0	20	6.65	6.65	6.65

*S, Subject; Pre, Pre assessment; Post, Post assessment; 3M, 3 Month-follow up; P, Paretic arm; NP, Non paretic arm; PROM, Passive Range of Motion; AROM, Active Range of Motion; P/NP, Paretic/Non Paretic ratio; W-II-V, Wrist; 2nd and 5th Metacarpophalangeal joints; II-V, 2nd and 5th Metacarpophalangeal joints; AVE, Average; CMSA-H, Chedoke-McMaster Stage of Hand section; UEFM, Upper Extremity Fugl-Meyer Assessment (Max score 66); SWMT, Cutaneous Sensory Touch Threshold using Semmes-Weinstein Monofilaments (normal detection threshold, 2.83 filament; 0.05 grams); NSA, Nottingham-Stereognosis (Sensory Impairment ≤ 19); BBT, Box and Blocks Test*.

### Activity measure scores

For the BBT, six out of eight subjects scored 0 at the pre-assessment evaluation, and six out of eight subjects increased at least one block at the post-assessment (see Table 3). The mean increase between pre- and post-assessment was 1.87 blocks. Nonparametric Friedman test revealed a statistically significant effect of intervention, χ^2^ = 10.38, *p* < 0.01. *Post-hoc* analysis with Wilcoxon signed-rank tests with Dunn-Sidak test revealed a significant increase from pre- to post-assessment (*p* < 0.05). However, there was no retention of these gains but with a significant BBT decrease at 3-month follow up compared to post-assessment (*p* < 0.05). Only one subject surpassed the minimal detectable change (MDC = 5.5 blocks) and reached the smallest real difference (SRD) (6 blocks) for BBT (see Table 3).

### Motor impairment clinical assessment scores

Similarly, the intervention had a significant effect on the active and passive ROMs, χ^2^ = 11.31, *p* < 0.05 and χ^2^ = 9.87, *p* < 0.05, respectively. *Post hoc* testing found a significant increase in the post-assessments at the level of *p* < 0.05. However, effects on the active and passive ROMs were not retained at the 3-month follow-up. Six out of eight subjects for AROM and seven subjects for PROM showed an improvement of at least 5° in one of the measured joints between pre- and post- assessments, which is the threshold for goniometer measurement error (ME) for the fingers (47). There was no main effect of intervention on grip strength (χ^2^ = 4.71, *p* = 0.09). Additionally, no significant changes were found in either UEFMA or CMSA-H.

### Sensory impairment clinical assessment scores

The intervention had a significant effect on the NSA scores (χ^2^ = 6.42, *p* < 0.05). Five out of eight participants showed an improvement of at least 10% from pre- to post-assessment in the NSA scores (see Table 3). It is worth noting that out of three participants, two showed pre-NSA scores >18, and thus could not have an improvement >10%, and the other participant showed a pre-NSA score = 18. There was no effect of intervention on Cutaneous Sensory Touch Thresholds using Semmes-Weinstein monofilaments.

Overall, after intervention, subjects showed significant improvements in the BBT scores, the PROM and AROM, and the stereognosis (NSA). At 3-month follow-up, improvements were not retained.

## Discussion

This study aimed to examine the impact of the reaching and grasping training aided by the ReIn-Hand device on UE function of chronic, severely impaired poststroke individuals. The ReIn-Hand device is unique in its ability to enable even severely impaired individuals with stroke to open the hand reliably regardless of proximal arm position and activation level of the shoulder abductor muscles (31). In this case series, eight individuals participated in a 7 week intervention using this device with clinical outcome measures taken at pre-, post-, and 3-month follow-up interventions. Given the small sample, this study served primarily as a pilot investigation for the clinical effectiveness of this intervention.

Our results show that in severely impaired individuals with poststroke hemiparesis, device-assisted reaching and grasping training may produce gains by reducing impairment and increasing activity levels. To determine the changes in activity level, specifically at manual dexterity and in UE function, participants were evaluated using the BBT. As the main outcome of the study, we found a significant increase from pre- to post-intervention assessment, but the gains were not maintained at 3-month follow-up. Specifically, six out of eight subjects showed an increase of at least one block. The increase in post-BBT scores may have been aided by the post-intervention gains in the AROM, since improvements in the ROMs, especially active, may contribute to improved upper extremity function (48, 49). In addition, gains in the ability to overcome the flexion synergy, that is, being able to open the hand while lifting the arm against gravity (while abducting the shoulder), might also have contributed to the significant gains on this assessment. The fact that individuals did not maintain gains at the 3-month follow-up could be due to the decreased use of the arm during ADLs, which may be possibly linked to learned nonuse, habit, or remaining impairments, thus resulting in decreased functional use of the paretic upper limb. The established MDC value for BBT outcome measure is 5.5 blocks per min (35) and the SRD is 6 blocks per min (50). One participant in our study was able to attain the MDC and the SRD scores between pre- and post-intervention assessments. The MCD value for BBT was established using data from 62 stroke subjects, whose BBT scores of the more affected hand at the first session were ≥1 block (mean ± SD = 23.1 ± 10.6). The authors reported an ME that was calculated to determine if the change score of an individual participant was above the 95% confidence level. Considering that six out of our eight subjects had a pre-BBT score equal to 0 (mean ± SD = 1.5 ± 4), our subjects were more impaired and would not have been qualified for the study to establish the MCD for the BBT. Our intervention induced a mean change of 1.87 in the BBT score, which is close to our calculated ME (=1.9). Moreover, our population was in a more chronic stage (median = 9 years) compared with the previous population that was used to establish the MDC for BBT (median = 8 months).

Active and passive ROM of the hand and wrist showed a significant increase from pre- to post- assessment. This improvement might possibly be due to the effect of FES on motor control (51, 52), joint ROMs (53), muscle tone (flexor hypertonicity induced) (54–56), and the synchronization between sensorimotor stimulation with muscle activity (57). However, these changes were not retained at the 3-month follow-up in this study.

Grip strength has been shown to influence ADLs (39, 58). The present results show no pre-to-post changes in grip strength (*p* = 0.09). The lack of significant increase in grip strength may be due to the fact that the stimulation exclusively facilitated hand opening (wrist/finger extensors) and was never aimed at finger/wrist flexors.

No significant changes were found in either UEFMA or CMSA-H between pretesting, posttesting, and follow-up testing. We think that these two assessments might not have enough resolution (59, 60) to detect small changes at the impairment level. Moreover, the length of the intervention (7 weeks) and the fact that there was limited progress in loading the UE (61, 62) during the intervention could have also been a reason for the lack of change.

Sensory deficits (tactile and stereognosis) are a common problem following stroke. Stereognosis requires the combination of many integrated primary sensory inputs (63). There is an association between somatosensory and motor impairments, somatosensory and UE activity limitations, and motor impairments and UE activity limitations, which increases with time after stroke (64). An intact sensorimotor network has been shown to be a prerequisite for purposeful arm use (64, 65) by allowing manipulation, coordination, and strength skills to be adapted to specific tasks (66). In our study, we found from the NSA that five out of eight subjects showed an improvement of at least 10%, and the other three participants had pre-NSA > = 18, thus having limited or no room for 10% improvement. Five participants were able to maintain the NSA gains at 3-month follow-up. We believe that the combination of FES with a functional task might have provided motor practice and tactile sensory feedback (i.e., touch, pressure) that is important for the acquisition of new skills (67) since any type of motor stimulation implies, to varying degrees, integration of sensory information (68). Furthermore, improvements in this stereognosis might also be partially explained by improvements in manual dexterity (69). We did not find significant changes in the Cutaneous Sensory Touch Threshold using Semmes-Weinstein Monofilaments. One of the reasons for this lack of significance may be a ceiling effect on this assessment. Five out of eight subjects scored a “normal detection threshold” (2.83 filament, 0.05 grams), which is the maximum possible score (43, 70) at pretest.

The underlying mechanisms for the improved hand/arm function from the observations are not fully understood. We did assess changes in grip strength and found no significant change. However, it is possible that that other strength changes might have occurred, such as an increase in shoulder abduction or an elbow extension. Therefore, we cannot fully assess if the adaptation was a result of increased muscle strength or true recovery. Instead, we measured intervention-induced cortical changes and reported an increased reliance on the ipsilesional hemisphere during hand opening following the intervention (27). Since motor recovery is typically reflected as a restoration in function of the neural tissues that were initially lost after injury (71), we believe that this may indicate that the motor improvement seen here is due to functional recovery to at least some extent. We do not believe that the clinical improvements reported in this study could have occurred spontaneously since all participants were in the chronic phase after stroke (average years post onset = 11.2) and were not participating in any other therapy.

## Potential limitations

Several limitations of this study should be noted. First, there was a small sample size that included a homogenous population. Second, the intervention study was limited to 7 weeks and 20–30 trials/session, whereas other studies have looked at interventions in this population for up to 12 weeks (72). Third, there were no self-reported measures or instruments that evaluated participation, so we cannot make conclusions about the effect of intervention from the patient's perspective. Fourth, there was no control group. Fifth, the carryover effect was only evaluated 3 months after the intervention, so we do not know when in that 3-month period the effect washed out and if subsequent training could have prevented it. The addition of multiple earlier follow-up evaluations could be used to determine when additional intervention should be scheduled to maintain the training effect. Sixth, UE stretching prior to and during the intervention could have been a confounding factor. However, a single session of stretching in general does not produce clinically important changes in joint ROM, pain, spasticity, or activity limitations (73–75). Although the long-term effects of passive stretching on joint ROM, spasticity, or activity limitations have not been reported yet (73–75), we believe stretching is not a stand-alone effective treatment for hand function in this population. Furthermore, the study lacked double blinding. Therefore, further research is needed in this specific population to examine the effectiveness and required dosage of this intervention, with larger samples sizes and a more heterogenous population in a randomized controlled trial.

In addition, the ReIn-hand device uses a triggered mode. Once triggered, an individual can choose to relax instead of continuing to try to open, since attempting to open can actually result in closing and diminishes the FES-generated opening in individuals with severe motor impairments (25). This can cause a slack effect. Currently, we do not have direct evidence to show the effect of the slack from subjects relying on the FES. Considering that this population cannot sufficiently open their paretic hands (six/eight individuals had a score in the BBT = 0 before the intervention), we focused on enhancing each individual's hand opening ability based on their voluntary control during the performance of a functional task that includes reaching and grasping. This action would provide desired synchronized proprioceptive and somatosensory feedback with motor tasks. Such synchronization is preferred since it increases Hebbian learning by strengthening the involved synapses (76) and acts as a signal for axonal sprouting after cortical lesions (77). Whether or not such slack will result in maladaptation is still unknown.

## Conclusions

These results suggest that using the ReIn-Hand device during functional reaching and grasping activities may contribute to improvements in gross motor function and stereognosis sensation of the paretic arm in individuals with moderate to severe impairment following chronic stroke.

## Clinical messages

- Task-specific training aided by the ReIn-Hand device might improve motor and sensory function in severely impaired chronic stroke.
- Further research is needed to assess the effectiveness of this intervention for improving clinical outcomes in randomized controlled trials.

## Ethics statement

This study was carried out in accordance with the recommendations of Northwestern University, institutional review board with written informed consent from all subjects. All subjects gave written informed consent in accordance with the Declaration of Helsinki. The protocol was approved by the name of committee.

## Author contributions

CC helped with the intervention design, participated in running the intervention, performed the clinical assessments, and was the primary author of the manuscript. KW participated in running the intervention, processed the data, and helped with manuscript writing. JD participated in running the intervention and helped with manuscript preparation. JS helped in designing the intervention and manuscript writing. JPAD helped with manuscript writing. JY was the primary designer of the intervention, participated in running the intervention, and helped with manuscript writing and editing.

### Conflict of interest statement

The authors declare that the research was conducted in the absence of any commercial or financial relationships that could be construed as a potential conflict of interest.
