# Corrigendum: Improving Hand Function of Severely Impaired Chronic Hemiparetic Stroke Individuals Using Task-Specific Training With the ReIn-Hand System: A Case Series

**DOI:** 10.3389/fneur.2019.01104

**Published:** 2019-10-17

**Authors:** Carolina Carmona, Kevin B. Wilkins, Justin Drogos, Jane E. Sullivan, Julius P. A. Dewald, Jun Yao

**Affiliations:** ^1^Department of Physical Therapy and Human Movement Sciences, Northwestern University, Chicago, IL, United States; ^2^Department of Biomedical Engineering, Northwestern University, Chicago, IL, United States; ^3^Department of Physical Medicine & Rehabilitation, Northwestern University, Chicago, IL, United States

**Keywords:** stroke, upper extremities, rehabilitation, functional electric stimulation (FES), hand function, task-practice

An author name was incorrectly spelled as “Carolina Camona.” The correct spelling is “Carolina Carmona.”

The authors apologize for this error and state that this does not change the scientific conclusions of the article in any way. The original article has been updated.

